# Experience-Dependent Plasticity to Visual Sequences in Mouse Anterior Cingulate Cortex Reflects Familiarity

**DOI:** 10.1523/JNEUROSCI.0508-25.2025

**Published:** 2025-10-29

**Authors:** Ayse Malci, Daniel P. Montgomery, Nishitha S. Hosamane, Keegan Whittle, Michael S. Sidorov

**Affiliations:** ^1^Center for Neuroscience Research, Children’s National Medical Center, Washington, DC 20010; ^2^University of Maryland College Park, College Park, Maryland 20742; ^3^Departments of Pediatrics and Pharmacology and Physiology, The George Washington University School of Medicine and Health Sciences, Washington, DC 20052

**Keywords:** Angelman syndrome, anterior cingulate cortex, experience-dependent plasticity, visual system

## Abstract

Anterior cingulate cortex (ACC) is a prefrontal area implicated in functions including cognitive control, attention, and prediction. Mouse ACC receives input from the visual system and uses visual information to direct behavior. While extensive work has described experience-dependent plasticity in mouse V1, less is known about how ACC itself adapts to visual experience. Our previous work demonstrated that visual sequences, presented across days, can drive plasticity in the timing of visually evoked responses in mouse ACC. However, it is not known whether this plasticity (“sequence plasticity”) reflects familiarity to the first stimulus in a sequence or expectation of subsequent stimuli—a distinction that is critically important for understanding its functional significance. We recorded visually evoked responses in awake, head-fixed female and male mice trained with visual sequences across days. Visual sequences drove plasticity in ACC, expressed through a change in response timing, that reflects familiarity to the first stimulus. In addition, experience-dependent plasticity could be induced using single-orientation stimuli. Together, these findings suggest that “sequence plasticity” in ACC does not in fact require sequences, but rather reflects a broader phenomenon that we term stimulus-specific response plasticity in timing (SRPT). Our prior work demonstrated that ACC plasticity is impaired in a mouse model of Angelman syndrome (AS). Here, AS model mice showed abnormal responses to familiar visual stimuli in ACC, despite normal plasticity in V1. Together, this work demonstrates how mouse ACC adapts to familiar visual stimuli and describes impaired ACC function in a mouse model of a neurodevelopmental disorder.

## Significance Statement

Our prior work established that familiar visual sequences can drive experience-dependent “sequence plasticity” in mouse anterior cingulate cortex (ACC). However, it is not known whether this plasticity reflects familiarity or expectation. Here, we show that sequence plasticity in ACC reflects familiarity, and is part of a broader phenomenon that can be driven by familiar visual stimuli other than sequences. In addition, our findings suggest impaired ACC function in a mouse model of the neurodevelopmental disorder Angelman syndrome. Overall, this work describes how mouse ACC adapts to encode familiar visual stimuli and demonstrates that this process is impaired in a mouse model of a neurodevelopmental disorder.

## Introduction

Anterior cingulate cortex (ACC) is a prefrontal subregion that plays a crucial role in complex functions such as cognitive control, attention, and prediction (Miller and Cohen, 2001; Le Merre et al., 2021). In mice, ACC is reciprocally connected with visual cortex, and ACC projections modulate a range of visually guided behaviors (Ahrlund-Richter et al., 2024). ACC itself is visually responsive in mice (Mohajerani et al., 2013; Hamm et al., 2021; Peters et al., 2022); however, our understanding of how visual experience modifies ACC circuits is limited relative to V1, which is often used as a model system.

In mouse V1, two related but distinct forms of experience-dependent plasticity can be driven by presenting awake, head-fixed mice with tightly controlled patterns of visual input. First, stimulus-specific response potentiation (SRP) is a phenomenon whereby repeated presentations of familiar stimulus across days drives an increase in the magnitude of visually evoked responses (Frenkel et al., 2006; Montgomery et al., 2021) across all cortical layers (Hayden et al., 2023). Second, spatiotemporal sequence potentiation, or sequence plasticity, can be induced in V1 by training mice using sequences of four ordered stimuli (Gavornik and Bear, 2014). Like SRP, V1 sequence plasticity is expressed by an increase in the magnitude of responses driven by the familiar sequence. However, V1 SRP and sequence plasticity differ both in their underlying mechanisms (Finnie et al., 2021; Sarkar et al., 2024) and in their likely behavioral significance. While V1 SRP reflects familiarity and behavioral habituation (Cooke et al., 2015), sequence plasticity is also thought to reflect temporal expectation (Price and Gavornik, 2022).

Our prior work demonstrated that in ACC, sequence plasticity is expressed through a change in the timing of visually evoked potentials (VEPs) driven by familiar sequences (Sidorov et al., 2020). This finding suggests that sequence plasticity is expressed differently in ACC (via changes in VEP timing) than in V1 (via changes in VEP magnitude). In this prior study, we trained mice using sequences of four unique stimuli, always presented in the same order (ABCD). Experience-dependent plasticity occurred in the timing of responses to stimulus B; thus, we hypothesized that ACC sequence plasticity may reflect the expectation that stimulus B follows stimulus A. This hypothesis is supported by work implicating ACC in visual expectation and prediction in other contexts (Fiser et al., 2016; Akam et al., 2021; Bastos et al., 2023; Cole et al., 2024). Alternatively, ACC sequence plasticity may reflect familiarity to stimulus A and occur regardless of whether an expected stimulus follows A in the sequence. If this alternative hypothesis were correct, it would suggest that presumed sequence plasticity in ACC is actually more akin to SRP, whereby plasticity reflects behavioral familiarity to a single stimulus. Here, we sought to explicitly evaluate whether ACC sequence plasticity reflects expectation of stimulus B or familiarity to stimulus A.

Understanding whether ACC encodes visual familiarity or expectation has particular relevance for autism and autism-like disorders. A theory for the development of autism suggests a failure of predictive processes and disrupted causality (Cannon et al., 2021). Indeed, individuals with autism show impairments in a visual sequence learning task and associated impairments in EEG event-related potential adaptation during the task (Thillay et al., 2016). Angelman syndrome (AS), an autism-like neurodevelopmental disorder, is associated with lack of speech, epilepsy, and motor and cognitive deficits resulting from loss of function mutations in the maternal copy of the *UBE3A* gene (Thibert et al., 2013). We previously showed that the *Ube3a^m−/p+^* AS mouse model lacks sequence plasticity in ACC, despite normal plasticity in V1 (Sidorov et al., 2020), and has deficits in visually guided attentional behavior (Negron-Moreno et al., 2022) that are likely ACC-dependent (Koike et al., 2016). Whether ACC plasticity reflects familiarity or expectation is important for understanding of sensory deficits in AS and autism, as disruptions in either process could lead to issues with recognizing familiar versus novel stimuli or developing expectations in sequential contexts.

## Materials and Methods

### Animals

All mice (C57BL/6J) were group housed on a 12 h light/dark cycle with *ad libitum* access to food and water. In all experiments, we used littermate controls, and experimenters were blind to genotype during experiments and data analysis. Littermates were randomly assigned to experimental groups. Equal ratios of adult [postnatal days (P) 80–120] male and female mice were used. Experimental AS model mice (*Ube3a^m−/p+^*) and wild-type (WT) littermates (*Ube3a^m+/p+^*) used in this study were generated by crossing female *Ube3a^m+/p−^* and male *Ube3a^m+/p+^* breeders. This common breeding scheme ensures that mothers have normal Ube3a protein expression in the brain. The original source of the AS mouse line (#016590, RRID:MGI:3694359) was Jackson Labs. Mice were genotyped by Transnetyx genotyping services. All protocols were approved by the Children's National Hospital Institutional Animal Care and Use Committee (IACUC). Each experiment used a separate cohort of animals, with no animals being reused across different experiments or figures. Experiments in Figures 5, 8, and 9 and Figure S5 compared *Ube3a^m−/p+^* mice with WT littermate controls. The rest of the experiments in this work were performed with WT mice.

### Chronic microelectrode implants

During stereotaxic survival surgeries, mice were anesthetized using isoflurane (4% induction, 1.6–2% maintenance) with administration of the local analgesic bupivacaine (0.25%) under the scalp. Following small craniotomies, tungsten microelectrodes (3 mm long for ACC and 2 mm long for V1, FHC, #30070) were implanted in right ACC (from bregma, in mm: A/P: +1.2, M/L: +0.5, D/V: −1.47) and bilaterally in binocular V1 (from bregma, in mm: A/P: −3.2, M/L: ±3.0, D/V: −0.48). A silver ground wire was placed in cerebellum. A steel headpost was attached for head fixation anterior to the ACC electrode, and dental cement (C&B Metabond, Parkell) was used to build a protective head cap securing all implants. During surgeries, body temperature was monitored and regulated using a far infrared warming pad with controller (Kent Scientific). For the relief of pain and inflammation, carprofen (5 mg/kg) was administered subcutaneously at the end of surgeries, and mice were let to recover on heating pad prior to returning their home cage.

### In vivo local field potential recordings

**Figure 1.**
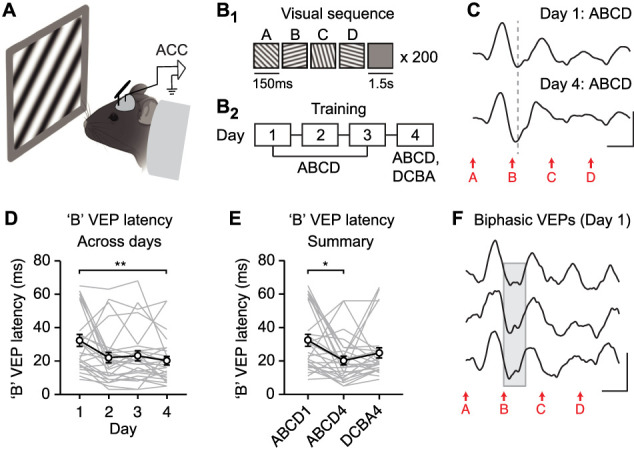
Visual sequences drive experience-dependent plasticity in ACC. ***A***, Head-fixed awake mouse views oriented sinusoidal gratings. ***B*_1_**, Presentation of visual sequences, each containing four elements in the same order (ABCD). ***B*_2_**, Mice were trained using familiar sequence ABCD across 4 d and also viewed novel sequence DCBA on Day 4. ***C***, Group average ACC VEPs on Day 1 and 4 of training. ***D***, Response latency to stimulus B across training (*n* = 27). ***E***, Response latency to second stimulus (B for ABCD sequence, C for DCBA sequence) as summary. ***F***, Day 1 ACC VEPs from three individual mice; the gray highlighted area indicates qualitatively biphasic “B” VEPs. Scale bars for all traces: 50 µV, 100 ms. *p < 0.05, **p < 0.01. Error bars represent SEM.

Following surgeries, mice recovered for at least 2 d in their home cage with *ad libitum* access to food and water. For all experiments, mice were habituated to the recording apparatus twice for 15 min per session on the day prior to experiments. During habituation and experiments, mice were awake, head-fixed, and body restrained in a dark, quiet environment and positioned facing a computer monitor displaying a gray screen (no active stimuli) at a distance of 20 cm (Fig. 1*A*). Prior to recordings, at the beginning of each session, mice were habituated for an additional 5 min. Local field potentials (LFPs) were recorded using the OmniPlex D Neural Data Acquisition System (Plexon) which amplified and digitized data at an acquisition rate of 40 kHz (downsampled to 1 kHz prior to analysis) and with 0.1 Hz high-pass and 200 Hz low-pass filtration.

### Visual stimuli

**Figure 2.**
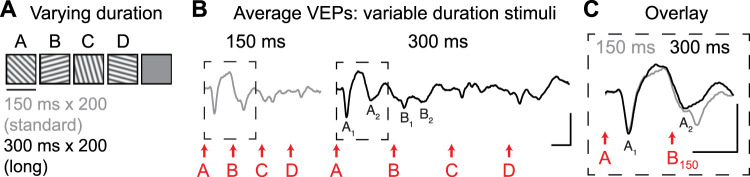
Extension of stimulus duration to 300 ms reveals two components of VEPs. ***A***, Mice viewed 400 total trials of ABCD sequences at varying durations (150 or 300 ms per element). ***B***, Average ACC VEPs with variable duration stimuli. Long-duration stimuli drove VEPs with two components. *n* = 9. ***C***, Overlay of average VEPs during the first 300 ms. Component A_2_ overlaps in time with a portion of the presumed “B” response to 150 ms stimuli. Scale bars for all traces: 20 µV, 100 ms.

**Figure 3.**
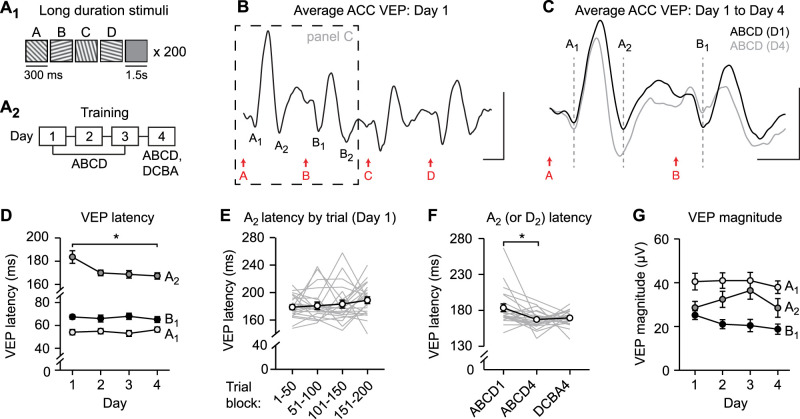
A2 latency is the locus of sequence plasticity in ACC. ***A*_1_**, Mice were trained using sequences of long-duration stimuli (ABCD, 300 ms per element). ***A*_2_**, Mice viewed sequence ABCD across 4 d and also viewed sequence DCBA on Day 4. ***B***, Group average ACC VEPs on Day 1 have two components (labeled as A_1_, A_2_, B_1_, and B_2_). ***C***, Group average VEPs on Day 1 (black) and Day 4 (gray) to sequence ABCD. The first ∼550 ms of traces are shown to emphasize A_1_, A_2_, and B_1_ response latencies (dashed rectangle in ***B***). Dashed lines indicate how latency is quantified for each VEP component. ***D***, Quantification of VEP latency across days of training (*n* = 22); asterisks represent a significant interaction based on two-way RM ANOVA. ***E***, Quantification of A_2_ VEP latency during the four blocks of 50 trials on Day 1. ***F***, Summary: response latency of A_2_ VEP component (or D_2_ for DCBA sequence) across Day 1 and Day 4. ***G***, VEP magnitude across training. Scale bars: ***B*** and ***C***, 25 µV, 100 ms. *p < 0.05. Error bars represent SEM.

**Figure 4.**
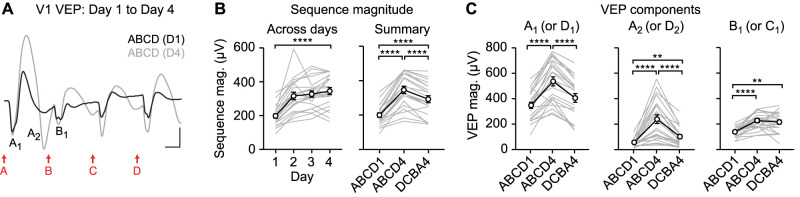
VEP magnitude potentiates with experience in V1. ***A***, Average V1 traces comparing ABCD1 and ABCD4. *n* = 21. ***B***, Sequence VEP magnitudes defined as the average of VEP magnitude following each stimulus ((A + B + C + D) / 4) are shown during training (left) and as a summary (right). ***C***, Sequence magnitude of A_1_, A_2_, and B_1_ VEP components as a summary (D_1_, D_2_, and C_1_ VEPs for DCBA sequence). Scale bars: ***A***, 100 µV, 100 ms. **p < 0.01, ****p < 0.0001. Error bars represent SEM.

**Figure 5.**
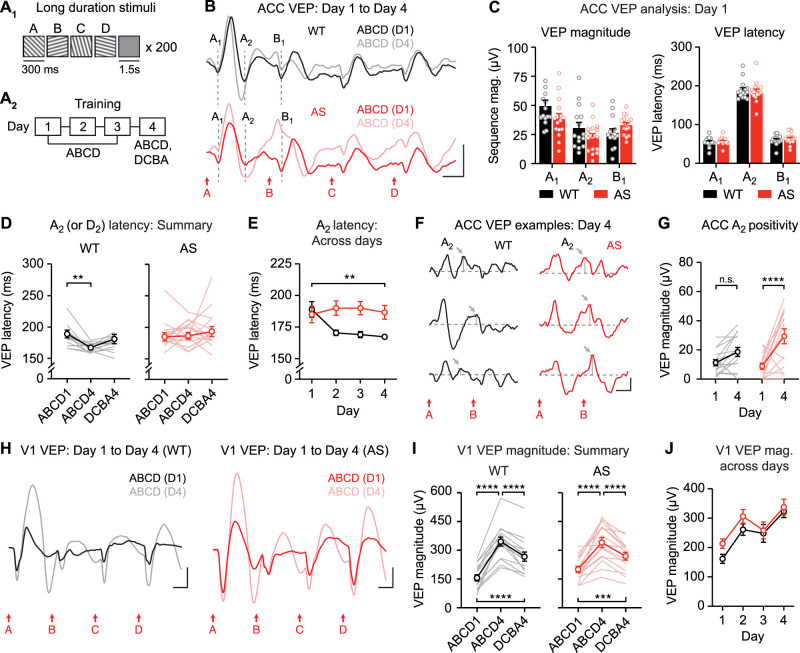
Sequence plasticity is altered in ACC, but unaltered in V1, in *Ube3a^m−/p+^* mice. ***A*_1_**, Wild-type (WT) and *Ube3a^m−/p+^* (AS) mice viewed visual sequences with 300 ms stimulus duration. ***A*_2_**, Mice viewed sequence ABCD across 4 d and both sequence ABCD and DCBA on Day 4. ***B***, Traces show group average VEPs in ACC on Day 1 and Day 4 for WT (top, black) and *Ube3a^m−/p+^* mice (bottom, red). WT: *n* = 15; AS: *n* = 16. Scale bars: 25 µV, 100 ms. Dashed lines indicate the timing of A_1_, A_2_, and B_1_ VEP components. ***C***, Magnitude and latency of VEP components A_1_, A_2_, and B_1_ on Day 1 in WT and *Ube3a^m−/p+^* mice. ***D***, Summary: response latency of A_2_ (or D_2_ for DCBA sequence) VEP component in WT (left) and *Ube3a^m−/p+^* mice (right) mice on Day 1 and Day 4. ***E***, Response latency of A_2_ VEP components in WT and *Ube3a^m−/p+^* mice across training. ***F***, Example ACC VEPs from individual mice on Day 4 of training. Gray arrows indicate A_2_ positivity. ***G***, A_2_ positivity on Day 1 and Day 4. ***H***, Traces show group average V1 VEPs on Day 1 and Day 4 in WT (left) and *Ube3a^m−/p+^* mice (right). WT: *n* = 15; AS: *n* = 16. Scale bars: 100 µV, 100 ms. ***I***, Summary of Day 1 and Day 4 in V1. ***J***, Sequence VEP magnitudes in V1 across days. **p < 0.01, ****p < 0.0001. Error bars represent SEM.

**Figure 6.**
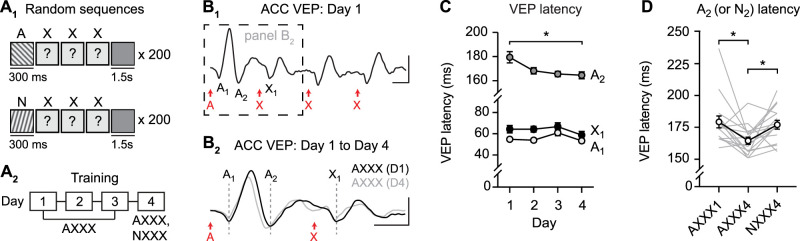
Sequence plasticity reflects familiarity to the first stimulus. ***A*_1_**, Mice viewed sequences where the first stimulus is consistent and the next three elements were pseudorandomly structured. ***A*_2_**, Mice viewed sequence AXXX across 4 d and also viewed sequence NXXX on Day 4, where N is a novel stimulus. ***B*_1_**, Group average ACC VEPs on Day 1 (*n* = 16). ***B*_2_**, Group average ACC VEPs during the first ∼560 ms of sequence presentation (dashed rectangle in ***B*_1_**) on Day 1 and Day 4. Dashed lines indicate latency of A_1_, A_2_, and X_1_ VEP components. ***C***, Response latency of VEP components across training; asterisks represent a significant interaction based on two-way RM ANOVA. ***D***, Summary of A_2_ latency (or N_2_ for NXXX sequence) on Days 1 and 4. Scale bars: ***B***, 25 µV, 100 ms. *p < 0.05. Error bars represent SEM.

**Figure 7.**
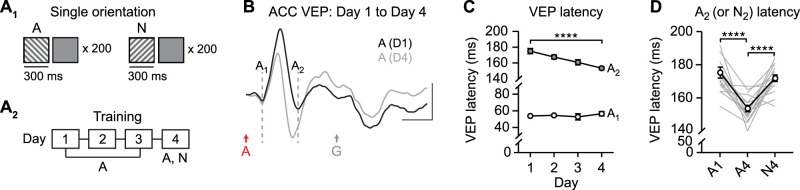
Training to individual stimuli drives plasticity in ACC response latency. ***A*_1_**, Mice viewed individual stimuli (A or N) with 300 ms duration, separated by gray screen periods. A, familiar; N, novel. ***A*_2_**, Training across 4 d. ***B***, Group average ACC VEPs on Day 1 (black) and Day 4 (gray) to familiar stimulus A (*n* = 16). Dashed lines indicate latency of A_1_ and A_2_ VEP components; G, gray. ***C***, Response latency of VEP components across training; asterisks represent a significant interaction based on two-way ANOVA. ***D***, Summary of A_2_ latency (or N_2_) on Days 1 and 4. Scale bars: ***B***, 25 µV, 100 ms. *p < 0.05, ****p < 0.0001. Error bars represent SEM.

**Figure 8.**
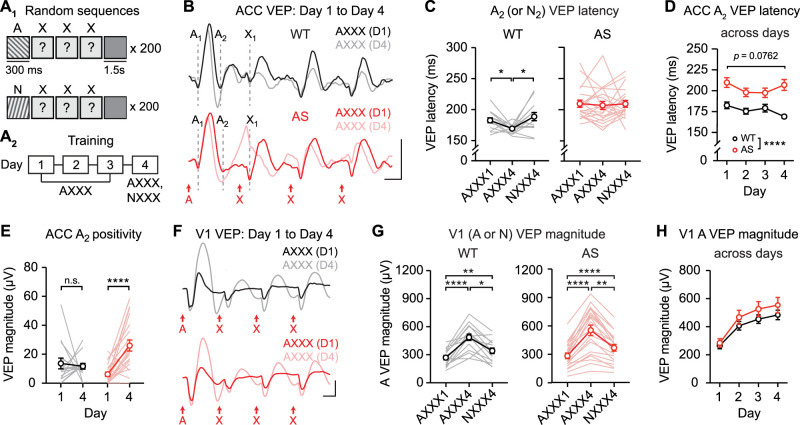
Plasticity driven by familiar visual stimuli is impaired in ACC, but unaltered in V1, in *Ube3a^m−/p+^* mice. ***A*_1_**, Wild-type (WT) and *Ube3a^m−/p+^* (AS) mice viewed pseudorandom sequences with 300 ms stimulus duration. ***A*_2_**, Mice viewed sequence AXXX across 4 d and both sequence AXXX and NXXX on Day 4 (N, novel). ***B***, Traces show group average VEPs in ACC on Day 1 and Day 4 for WT (top, black) and *Ube3a^m−/p+^* mice (bottom, red). WT: *n* = 15; AS: *n* = 13. Dashed lines indicate timing of A_1_, A_2_, and X_1_ VEP components. Scale bars: 25 µV, 100 ms. ***C***, Response latency of A_2_ (or N_2_) VEP components in WT (left) and *Ube3a^m−/p+^* mice (right) as a summary. ***D***, Comparison of response latency of A_2_ VEP components in WT and *Ube3a^m−/p+^* mice across training. ***E***, A_2_ positivity on Day 1 and Day 4. ***F***, Traces display group average V1 VEPs on Day 1 and Day 4 for WT (top) and *Ube3a^m−/p+^* mice (bottom). WT: *n* = 15; AS: *n* = 13. Scale bars: 100 µV, 100 ms. ***G***, The magnitude of VEPs to the first stimulus (A or N) in WT (left) and *Ube3a^m−/p+^* mice (right) as a summary. ***H***, The A VEP magnitudes in V1 across days. *p < 0.05, **p < 0.01, ****p < 0.0001. Error bars represent SEM.

**Figure 9.**
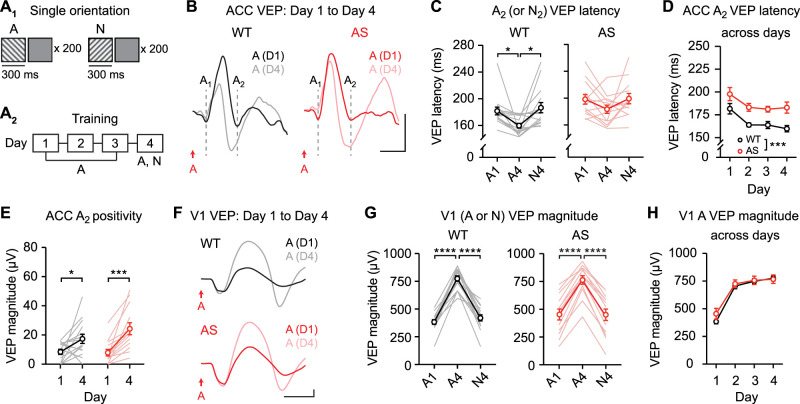
Plasticity driven by single-orientation stimuli is impaired in ACC, but unaltered in V1, in *Ube3a^m−/p+^* mice. ***A*_1_**, Mice viewed 200 presentations of single-orientation stimuli (300 ms duration). ***A*_2_**, Mice viewed stimulus A across 4 d and both familiar (A) and novel (N) stimuli on Day 4. ***B***, Traces show group average VEPs in ACC on Day 1 and Day 4 for WT (left, *n* = 14) and *Ube3a^m−/p+^* mice (right, *n* = 20). Dashed lines indicate timing of A_1_ and A_2_ VEP components. Scale bars: 25 µV, 100 ms. ***C***, Response latency of A_2_ (or N_2_) VEP component in WT (left) and *Ube3a^m−/p+^* mice (right) as a summary. ***D***, Response latency of A_2_ VEP components in WT and *Ube3a^m−/p+^* mice across training. ***E***, A_2_ positivity on Day 1 and Day 4. ***F***, Traces show group average V1 VEPs on Day 1 and Day 4 for WT (top) and *Ube3a^m−/p+^* mice (bottom). WT: *n* = 16; AS: *n* = 20. Scale bars: 100 µV, 100 ms. ***G***, The magnitude of VEPs to stimulus 1 (A or N) in WT and *Ube3a^m−/p+^* mice as a summary. ***H***, The A VEP magnitudes in V1 across days. *p < 0.05, ***p < 0.001, ****p < 0.0001. Error bars represent SEM.

**Figure 10.**
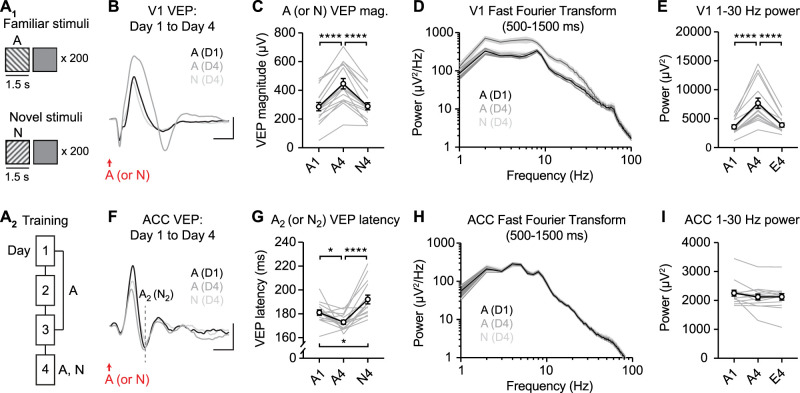
Low-frequency oscillations are increased in V1, but unchanged in ACC, following training with single-orientation stimuli. ***A*_1_**, Mice viewed single-orientation stimuli (A or N) with 1.5 s duration, separated by gray screen periods. A, familiar; N, novel. ***A*_2_**, Training across 4 d. ***B***, Group average V1 VEPs on Day 1 (black) and Day 4 (dark gray) to familiar stimulus A and Day 4 to novel stimulus N (light gray). ***C***, The magnitude of VEPs to stimuli (A or N) in V1 as a summary. ***D***, Fast Fourier transform (FFT) of V1 LFPs calculated in the stationary period following the VEP (between 500 and 1,500 ms after stimulus onset) shows power spectra for familiar and novel stimuli. ***E***, The quantification of V1 1–30 Hz power to stimuli (A or N) as a summary. ***F***, Group average ACC VEPs on Day 1 (black) and Day 4 (dark gray) to familiar stimulus A and Day 4 to novel stimulus N (light gray). Dashed line indicates latency of A_2_ (or N_2_) VEP component. ***G***, Response latency of A_2_ (or N_2_) VEP components as a summary. ***H***, FFT of ACC LFPs in the stationary period following the VEP shows no change is oscillatory power for familiar (A) and novel (N) stimuli. ***I***, The quantification of ACC 1–30 Hz power to stimuli (A or N) as a summary. Traces display 600 ms following stimulus onset. *n* = 15. Scale bars: ***B***, 100 µV, 100 ms; in ***F***, 25 µV, 100 ms. *p < 0.05, ****p < 0.0001. Error bars represent SEM.

Visual stimuli known to reliably evoke VEPs in both ACC and V1 (Gavornik and Bear, 2014; Sidorov et al., 2020) were generated using custom software written in MATLAB (MathWorks) with the Psychtoolbox extension (http://psychtoolbox.org/, https://github.com/jeffgavornik/VEPStimulusSuite). Each stimulus was an oriented sinusoidal grating with 100% contrast and 0.05 cycles/degree spatial frequency. Details about the visual stimuli used for each experiment are listed in Table S1. For experiments in Figures 1–5, visual sequences consisted of four consecutive stimuli (A, 45°; B, 105°; C, 15°; D, 75°), and each sequence was divided by a 1.5 s gray screen period. Each session had 200 trials, divided into four blocks of 50 trials with 30 s gray screen between blocks (Gavornik and Bear, 2014; Sidorov et al., 2020). Where noted (Figs. 1, 3–5), mice were trained using 200 trials of familiar sequence ABCD across 4 d, with one session per day. On Day 4, mice were presented with 200 trials of familiar sequence ABCD and 200 trials of novel sequence DCBA immediately after ABCD. Sequence DCBA represents familiar elements, but presented in a novel order, and was used to evaluate the extent to which plasticity was specific to the familiar sequence (Gavornik and Bear, 2014; Sidorov et al., 2020). For experiments in Figure 1, all visual stimuli had a duration of 150 ms. Sequence ABCD was presented 200 times per day across 4 d, with DCBA also presented 200 times on Day 4. For experiments in Figure 2, mice viewed 200 trials of sequence ABCD with 150 ms stimulus duration and 200 trials of sequence ABCD with 300 ms stimulus duration in a single session. For experiments in Figures 3–5, mice viewed 200 trials of sequence ABCD (300 ms stimulus duration) per day across 4 d, with DCBA (300 ms duration) also presented 200 times on Day 4. For experiments in Figures 6, 8, mice were trained across 4 d with 200 trials of sequence AXXX (300 ms stimulus duration) per day. Here, element A (45°) was consistent, and the rest of the sequences (XXX) were pseudorandom combinations of B (105°), C (15°), D (75°), and E (135°). There was no repetition of any element (e.g., ADDB) in a sequence. On Day 4, in addition to sequence AXXX, mice also viewed 200 trials of novel sequence NXXX (novel N: 165°). For experiments in Figures 7 and 9 and Figure S7, mice did not view sequences. In Figures 7 and 9, mice viewed 200 trials (4 × 50) of a single-orientation visual stimulus (A: 45°; 300 ms duration) followed by 1.5 s of gray screen across 4 d of training. On Day 4, mice also viewed 200 trials of a novel element (N: 135°; 300 ms duration) followed by 1.5 s of gray screen. In Figure S6, half of mice were trained with 200 trials of sequence ABCD (300 ms stimulus duration) while the other half were trained with sequence AXXX (300 ms) across 4 d. Figure S7 used a standard protocol for inducing SRP in V1 (Frenkel et al., 2006; Cooke et al., 2015; Chaloner and Cooke, 2022): 4 d of training to a familiar stimulus (A: 45°) that phase reverses at 2 Hz (400 total phase reversals; 4 × 100 with 30 s gray screen break). There was no gray screen in between phase reversals. On Day 4, mice also viewed 400 total phase reversals of a novel orientation (N: 135°). For experiments in Figure 10, mice were trained across 4 d with 200 trials of single-orientation stimulus (A: 45°; 1.5 s duration), followed by 1.5 s of gray screen. On Day 4, mice also viewed 200 trials of a novel element (N: 135°; 1.5 s duration) followed by 1.5 s of gray screen. In all experiments, mice were experimentally naive on Day 1.

### VEP analysis

Visually evoked potentials (VEPs) were calculated by averaging evoked LFP responses to all 200 trials within a single session in either ACC or V1 (Porciatti et al., 1999; Frenkel et al., 2006; Gavornik and Bear, 2014). VEPs were analyzed from each session using custom MATLAB software (https://github.com/jeffgavornik/VEPAnalysisSuite). VEP magnitude was calculated as the voltage difference between the peak negativity and peak positivity following stimulus presentation. Response latency was calculated as the time of the maximal negative-going peak following stimulus presentation as described previously (Gavornik and Bear, 2014; Sidorov et al., 2020). Where noted, response latency and/or VEP magnitude was calculated for multiple components of the VEP (Figs. 3–9). Here, we use the shorthand “A_1_” and “A_2_” to refer to the N1 and N2 components of the VEP driven by stimulus A, respectively. In V1, “sequence magnitude” was defined as an average of the VEP magnitude driven by each stimulus in the sequence [(A + B + C + D) / 4; Figs. 4, 5; Gavornik and Bear, 2014). In Figures 8 and 9, V1 VEP magnitude was quantified from the first VEP response driven by stimulus A. As V1 was recorded bilaterally, results from two hemispheres were averaged for each mouse. Prior to averaging VEPs, there was no significant variance between hemispheres in V1 data either in WT or *Ube3a^m−/p+^* mice (Fig. S1). In case of poor signal detected in one hemisphere, only data from the unaffected hemisphere were considered. To determine plasticity across training, analyzed VEPs were compared between sessions (from Day 1 to Day 4; Figs. 1*D*, 3*D*, 4*B*, 6*C*, 7*C*; Fig. S7*C*). To evaluate whether plasticity was specific to the trained stimuli, familiar versus novel stimuli were compared on Day 4 (Figs. 1*E*, 3*F*, 4*B*,*C*, 5*D*,*I*, 6*D*, 7*D*, 8*C*,*G*, 9*C*,*G*, 10*C*,*G*; Fig. S7*D*). To determine whether plasticity occurs within a single recording session, A_2_ response latency across 200 trials was quantified in four blocks of 50 trials (trials 1–50, 51–100, 101–150 and 151–200; Fig. 3*E*, Fig. S3*B*). ACC VEPs in trials fewer than 50 trials were not quantifiable due to a low signal-to-noise ratio (Fig. S2). To determine the amount of ACC plasticity in A_2_ VEP latency, we subtracted response latency on Day 1 from that on Day 4 (Fig. S6*C*). In WT and *Ube3a^m−/p+^* mice, we quantify the positive-going component of the A_2_ VEP component (Figs. 5*G*, 8*E*, 9*E*) by measuring the positive deflection of this VEP relative to the voltage at the onset of stimulus A (schematic in Fig. 5*F*).

### Oscillatory power analysis

Continuous LFP recordings were high-pass filtered using a ninth-order Butterworth filter with a 0.5 Hz cutoff to remove slow drifts. Spectral power was computed using fast Fourier Transform (FFT) applied to a 1 s segment of the local field potential beginning 0.5 s after stimulus onset, a window chosen to avoid early evoked transients and ensure relative stationarity of the signal, an approach consistent with previous work (Chalk et al., 2010; Hayden et al., 2021). Power was calculated from the squared magnitude of the complex Fourier coefficients. The 1 s analysis window provided a frequency resolution of 1 Hz. Power spectra from 1 to 100 Hz were computed separately for each trial and electrode, and trial-averaged spectra were used to quantify power from 1 to 30 Hz.

### Statistical analysis

Statistical analysis was performed using GraphPad Prism version 10.2.2. The results of all statistical tests are listed in Table S2. To evaluate plasticity across days or across trial blocks in wild-type mice, we used a one-way repeated-measures (RM) ANOVA (Figs. 1*D*, 3*E*, 4*B*, 10*C*,*E*,*G*,*I*; Figs. S3*B*, S7*C*). To evaluate if plasticity was specific to the trained stimuli, we used one-way RM ANOVA (Figs. 1*E*, 3*F*, 4*B*,*C*, 5*D*,*I*, 6*D*, 7*D*, 8*C*,*G*, 9*C*,*G*, 10*C*,*G*; Figs. S3*B*, S7*D*). To evaluate plasticity in multiple VEP components (e.g., A_1_, A_2_, B_1_) across days, we used two-way RM ANOVA (with VEP component and session as factors; Figs. 3*D*,*G*, 6*C*, 7*C*; Fig. S4). Two-way RM ANOVA was also used to compare V1 VEPs across hemispheres (Fig. S1*B*,*C*) and plasticity in ABCD and AXXX groups (Fig. S6*B*). To evaluate plasticity in two genotypes (WT vs *Ube3a^m−/p+^*) across days, data were analyzed using two-way RM ANOVA (with genotype and session as factors; Figs. 5*E*,*G*,*J*, 8*D*,*E*,*H*, 9*D*,*E*,*H*; Fig. S5*B*). Post hoc tests were used when there was a statistically significant main effect. For plasticity studies, Dunnett's post hoc test was used to compare each day to baseline (Day 1) and Tukey's multiple-comparison post hoc test was used to compare all days of training with each other where applicable. Šidák's post hoc test was used to compare WT and *Ube3a^m−/p+^* genotypes across days (Figs. 5*E*, 8*D*, 9*D*). Unpaired *t* tests were also used when comparing two conditions as appropriate (Fig. 5*C*; Figs. S6*C*, S7*E*). In all experiments, *n* represents the total number of mice per group. All data are presented as mean ± SEM. Statistical significance is denoted as *p < 0.05, **p < 0.01, ***p < 0.001, ****p < 0.0001.

## Results

### Experience-dependent plasticity to visual sequences in ACC is expressed through a change in the timing of evoked responses

Before formally testing whether ACC sequence plasticity reflects expectation or familiarity, we first aimed to replicate our prior finding that this form of plasticity is expressed through a change in VEP timing. Using a protocol similar to previous work (Gavornik and Bear, 2014; Sidorov et al., 2020), we recorded and quantified ACC VEPs in awake, head-fixed mice viewing sequences of four ordered visual stimuli (ABCD; A, 45°; B, 105°; C, 15°; D, 75°) across 4 consecutive days (Fig. 1*A*–*C*). On Day 4, mice also viewed the novel sequence DCBA. As previously reported, the latency of ACC VEPs to expected stimulus “B” decreased with experience (Fig. 1*D*, one-way RM ANOVA: main effect of session: *F*_(1.803,46.88)_ = 6.133, *p* = 0.0056). VEP latency was not statistically different across Days 2–4 (all post hoc tests reported in Table S2), suggesting that the largest changes in latency occur between Day 1 and Day 2. Sequence plasticity was partially but not fully specific to trained sequence ABCD (Fig. 1*E*, main effect of session: *p* = 0.0092, post hoc ABCD1 vs ABCD4: *p* = 0.0163, ABCD4 vs DCBA4: *p* = 0.3631, ABCD1 vs DCBA4: *p* = 0.1459). Overall, these findings are generally consistent with our prior work. However, interpretations were complicated by a surprising observation: Day 1 “B” VEPs appeared to be qualitatively biphasic in a subset of mice (Fig. 1*F*, 20/27 total). This finding was surprising because on Day 1, mice are experimentally naive and we would not expect plasticity to have occurred. Yet, Day 1 VEPs looked qualitatively as though plasticity had already occurred, with “B” VEP latencies ranging from ∼10–40 ms instead of the ∼60 ms latency expected on Day 1 (Sidorov et al., 2020). One possible explanation for this result is that plasticity has indeed occurred within Day 1 recordings, and averaging all 200 trials across the session results in a biphasic VEP. To address this possibility, we compared “B” VEP latency across the four blocks of 50 trials on Day 1. There was no change in “B” VEP latency across Day 1 blocks (Fig. S3), suggesting that the observed biphasic Day 1 VEPs indeed represent a true baseline and do not reflect within-session plasticity. Understanding the nature of Day 1 “B” VEPs is critically relevant to our central question of whether sequence plasticity reflects expectation of stimulus B or familiarity to stimulus A. One potential explanation of this phenomenon is that the second component of the “B” VEP, with ∼60 ms latency, is driven by stimulus B, while the first component, with ∼10–40 ms latency, is instead actually a secondary negativity (N2) driven by stimulus A. If so, this might suggest that plasticity in the first component reflects familiarity to A and not expectation of B. To test this question directly, we used stimuli with long stimulus durations, enabling separation of “A” and “B” responses in time.

### Long-duration stimuli resolve two VEP components and are better suited to evaluate plasticity in ACC

To test the hypothesis that presumed “B” VEPs are actually driven by both stimulus A and stimulus B, we extended the duration of visual stimuli from 150 to 300 ms. Mice viewed 400 total sequences in a single session: half with a standard stimulus duration of 150 ms and half with a long stimulus duration of 300 ms (Fig. 2*A*). Long-duration stimuli allowed for resolution of two distinct components to VEPs driven by both stimulus A (A_1_, A_2_) and stimulus B (B_1_, B_2_; Fig. 2*B*) in all mice tested. “Slow” VEP component A_2_ had a latency of ∼160–190 ms following stimulus A onset. The timing of this A_2_ response is consistent with the first component of the biphasic presumed “B” VEP driven by 150 ms stimuli (Fig. 2*C*). Thus, the biphasic response to 150 ms stimuli (Fig. 1*E*) likely reflects a composite A_2_ and B_1_ response occurring on overlapping timescales (Fig. 2*C*). The plasticity observed in Figure 1 could therefore reflect either a change in A_2_ or in B_1_. However, 150 ms stimuli are unable to sufficiently resolve A_2_ and B_1_ in time.

We next used long-duration (300 ms) stimuli to test whether plasticity occurs in VEP component A_2_ or B_1_ (Fig. 3*A–C*). Across 4 d of training with familiar sequences, there was a significant decrease in A_2_ VEP latency but not B_1_ or A_1_ VEP latency (Fig. 3*D*, two-way RM ANOVA: interaction: *F*_(6,189)_ = 2.840, *p* = 0.0114; post hoc for A_2_ Day 1 vs Day 4: *p* = 0.0329, A_1_ Day 1 vs Day 4: *p* = 0.8526, B_1_ Day 1 vs Day 4: *p* = 0.5964). There was no statistically significant change in A_2_ VEP latency across Days 2–4 (all post hoc tests reported in Table S2), suggesting that the largest changes in latency occur between Day 1 and Day 2. As with short duration sequences, there was no change in A_2_ VEP latency within Day 1 (Fig. 3*E*, one-way RM ANOVA: main effect of trial block: *F*_(2.408,50.57)_ = 0.9820, *p* = 3,944). A summary of plasticity over days is shown in Figure 3*F*, where on the final test day changes were not fully specific to trained sequence ABCD (one-way RM ANOVA: main effect of session: *F*_(1.741,36.56)_ = 5.324, *p* = 0.0121, post hoc ABCD1 vs ABCD4: *p* = 0.0324, ABCD4 vs DCBA4: *p* = 0.8754, ABCD1 vs DCBA4: *p* = 0.0628). There was no change in the magnitude of ACC VEP components with experience (Fig. 3*G*, main effect of session: *F*_(2.808,176.9)_ = 1.997, *p* = 0.1203). The magnitude and latency of other VEP components (B_2_, C_1_, C_2_, D_1_, D_2_) did not change with experience (Fig. S4).

In the same cohort of mice, we also recorded and quantified VEPs from V1. We hypothesized that sequence plasticity in V1, which is expressed by an increase in VEP magnitude (Gavornik and Bear, 2014), could also be driven using long-duration stimuli. Long-duration stimuli indeed drove sequence plasticity in V1 that was expressed through an increase in VEP magnitude (Fig. 4*A*,*B*, V1 VEP magnitude across days: one-way RM ANOVA: main effect of session: *F*_(2.849,56.98)_ = 48.03, *p* < 0.0001) and was partially specific to the trained sequence (Fig. 4*B*, summary: main effect of session: *F*_(1.144,28.79)_ = 92.05, *p* < 0.0001, post hoc ABCD1 vs ABCD4: *p* < 0.0001, ABCD4 vs DCBA4: *p* < 0.0001, ABCD1 vs DCBA4: *p* < 0.0001). Plasticity in V1 occurred to all elements of the sequence and was not specific to VEP component A_2_ (Fig. 4*C*, left, one-way RM ANOVA: main effect of session: *F*_(1.347,28.28)_ = 41.95, *p* < 0.0001, center: *F*_(1.144,24.02)_ = 33.71, *p* < 0.0001, right: *F*_(1.985,41.68)_ = 25.84, *p* < 0.0001). Overall, these results demonstrate that ACC encodes sequence plasticity in timing of the A_2_ VEP, unlike V1, where plasticity is expressed by an increase in the magnitude of VEPs overall. Because ACC sequence plasticity occurs in the A_2_ VEP prior to the presentation of stimulus B, this suggests that plasticity may reflect familiarity to A rather than expectation of B.

### Long-duration stimuli reveal the locus of impaired experience-dependent plasticity in *Ube3a^m−/p+^* mouse ACC

Previously we reported that ACC sequence plasticity is impaired in the *Ube3a^m−/p+^* mouse model of the neurodevelopmental disorder AS (Sidorov et al., 2020). To test the hypothesis that the A_2_ VEP is the locus of impaired plasticity in *Ube3a^m−/p+^* mice, we evaluated plasticity using long-duration sequences in *Ube3a* mutants and littermate controls (Fig. 5*A*,*B*). Day 1 baseline VEPs did not differ between genotypes (Fig. 5*C*, left, unpaired *t* test for A_1_ magnitude: *t*_(29)_ = 1.690, *p* = 0.1017; A_2_ magnitude: *t*_(29)_ = 1.437, *p* = 0.1613; B_1_ magnitude: *t*_(29)_ = 1.712, *p* = 0.0975; right, A_1_ latency: *t*_(29)_ = 0.4104, *p* = 0.6846; A_2_ latency: *t*_(29)_ = 0.4393, *p* = 0.6637; B_1_ latency: *t*_(29)_ = 0.7105, *p* = 0.4831). Wild-type ACC showed sequence plasticity (change in A_2_ VEP latency) that was partially specific to the trained sequence (Fig. 5*D*, left, one-way RM ANOVA: main effect of session: *F*_(1.728,24.20)_ = 4.149, *p* = 0.0331, post hoc ABCD1 vs ABCD4: *p* = 0.0069, ABCD4 vs DCBA4: *p* = 0.2488, ABCD1 vs DCBA4: *p* = 0.6166). However, A_2_ VEP latency was unchanged with experience in *Ube3a^m−/p+^* ACC (Fig. 5*D*, right, *F*_(1.595,23.48)_ = 0.4492, *p* = 0.5961). A direct comparison between genotypes revealed a statistically significant group difference in plasticity over days (Fig. 5*E*, two-way RM ANOVA: interaction: *F*_(3,87)_ = 5.090, *p* = 0.0027). In addition, the shape of the group-averaged A_2_ VEP appeared to be qualitatively different in *Ube3a^m−/p+^* mice on Day 4 (Fig. 5*B*): the positivity following A_2_ was especially prominent. This qualitative finding was observed in individual *Ube3a^m−/p+^* recordings and was not an artifact of averaging across animals (Fig. 5*F*). Quantitative analysis of A_2_ VEP positivity revealed a group difference (Fig. 5*G*, two-way RM ANOVA: interaction: *F*_(1,28)_ = 4.718, *p* = 0.0385), reflected as an increased in magnitude in *Ube3a^m−/p+^* mice (post hoc *Ube3a^m−/p+^* Day 1 vs Day 4: *p* < 0.0001), but no change in WT mice (WT Day 1 vs Day 4: *p* = 0.1782). A_1_ VEP magnitude did not differ between WT and *Ube3a^m−/p+^* mice across training (Fig. S5).

In the same mice, V1 plasticity occurred and was specific to the trained sequence in both WT and *Ube3a^m−/p+^* mice (Fig. 5*H*,*I*, left, one-way RM ANOVA: main effect of session: *F*_(1.509,21.13)_ = 82.17, *p* < 0.0001, right, one-way RM ANOVA: main effect of session: *F*_(1.162,16.27)_ = 36.90, *p* < 0.0001). There was no genotype difference in sequence plasticity in V1 (Fig. 5*J*, two-way RM ANOVA: main effect of session: *F*_(1.750,40.26)_ = 35.12, *p* < 0.0001, main effect of genotype: *F*_(1,23)_ = 1.326, *p* = 0.2614, interaction: *F*_(3,69)_ = 0.9278, *p* = 0.4321). Taken together, these results confirm our prior finding that sequence plasticity in *Ube3a^m−/p+^* mice is impaired in ACC and is normal in V1 (Sidorov et al., 2020). Using long-duration stimuli, we demonstrate that in ACC, the locus of this impaired plasticity is the A_2_ VEP component and that the A_2_ VEP component is broadly disrupted in *Ube3a^m−/p+^* ACC following sequence training.

### ACC sequence plasticity reflects familiarity to the first stimulus

To directly test the hypothesis that ACC sequence plasticity reflects familiarity, we asked whether plasticity in A_2_ VEP latency occurs in the absence of a consistent expected stimulus B. We trained WT mice using pseudorandom sequences always starting with stimulus A but followed by three pseudorandom orientations (“AXXX”; Fig. 6*A*). As expected, this protocol drove ACC VEPs with A_1_ and A_2_ components, as well as a “B_1_-equivalent” component that we label “X_1_” (Fig. 6*B*). With training to pseudorandom sequences across days, there was significant plasticity in the A_2_ VEP latency (Fig. 6*C*, two-way RM ANOVA: interaction: *F*_(6,135)_ = 2.794, *p* = 0.0136, post hoc A_2_ Day 1 vs A_2_ Day 4: *p* = 0.0262). There was no plasticity in either A_1_ VEP latency (Fig. 6*C*, post hoc A_1_ Day 1 vs A_1_ Day 4: *p* = 0.5580) or X_1_ VEP latency (Fig. 6*C*, post hoc X_1_ Day 1 vs X_1_ Day 4: *p* = 0.5715). There was no change in A_2_ VEP latency across Days 2–4 (post hoc tests reported in Table S2), again suggesting that the largest changes in latency occur between Day 1 and Day 2. Plasticity in the A_2_ VEP latency was specific to familiar stimulus A and did not generalize to sequences starting with the novel stimulus “N” (Fig. 6*D*, one-way RM ANOVA: main effect of session: *F*_(1.894,28.41)_ = 5.692, *p* = 0.0092, post hoc AXXX1 vs AXXX4: *p* = 0.0257, AXXX1 vs NXXX4: *p* = 0.8951, AXXX4 vs NXXX4: *p* = 0.0213). In a separate cohort, AXXX-trained mice and ABCD-trained mice showed a similar degree of plasticity in their ACC A_2_ VEPs (Fig. S6). Together, these results demonstrate that plasticity in A_2_ occurs even when there is no expectation of B following A. These findings suggest that sequence plasticity reflects familiarity to stimulus A and not expectation of stimulus B.

### Familiarity without sequences is sufficient to drive plasticity in ACC

We next reasoned that if sequence plasticity indeed reflects familiarity to stimulus A, then this plasticity would occur to stimulus A regardless of whether A is followed by other elements of a sequence. To test this hypothesis, mice underwent training with 200 trials of a single stimulus at the same orientation (A: 45°) over 4 consecutive days (Fig. 7*A*). As with sequence presentations, all individual stimuli were followed by a 1.5 s gray screen. As expected, stimulus A drove biphasic VEPs with components A_1_ and A_2_ (Fig. 7*B*). With experience across days, there was plasticity in the latency of VEP component A_2_ (Fig. 7*B*,*C*, two-way RM ANOVA: interaction: *F*_(3,90)_ = 7.954, *p* < 0.0001, A_2_ VEP latency: post hoc Day 1 vs Day 4: *p* < 0.0001) but not VEP component A_1_ (Fig. 7*C*, post hoc Day 1 vs Day 4: *p* = 0.7109). In this experiment, we did observe significant plasticity beyond Day 2 (post hoc tests in Table S2). Plasticity in A_2_ VEP latency was specific to the trained stimulus and did not generalize to novel stimulus N (Fig. 7*D*, one-way RM ANOVA: main effect of session: *F*_(1.863,27.94)_ = 27.47, *p* < 0.0001, post hoc A1 vs A4: *p* < 0.0001, A1 vs N4: *p* = 0.4614, A4 vs N4: *p* < 0.0001). These results demonstrate that plasticity reflecting familiarity can be driven by single-orientation stimuli and does not require sequences beyond this initial stimulus. However, a classical V1 SRP training protocol using phase reversals of a single orientation (with no gray screens separating stimuli) was insufficient to evaluate plasticity in ACC, as VEPs were substantially smaller and the A_2_ component was not reliably detected (Fig. S7). We conclude that the modified single-orientation stimulus protocol used in Figure 7 is better suited than phase reversals for ACC recordings.

### The A_2_ VEP component is disrupted in *Ube3a^m−/p+^* ACC across plasticity protocols

Because the A_2_ VEP component was impaired in *Ube3a^m−/p+^* ACC following training to visual sequences (Fig. 5), we next evaluated the consistency of this impairment across plasticity protocols. First, training with pseudorandom sequences (Fig. 8*A*,*B*) drove stimulus-specific plasticity in A_2_ VEP latency in WT mice (Fig. 8*C*, left, one-way RM ANOVA: main effect of session: *F*_(1.460,18.99)_ = 4.961, *p* = 0.0268), but not *Ube3a^m−/p+^* mice (Fig. 8*C*, right, one-way RM ANOVA: main effect of session: *F*_(1.886,35.84)_ = 0.1017, *p* = 0.8933). A direct comparison between genotypes revealed an increase in A_2_ VEP latency across days in *Ube3a^m−/p+^* mice (Fig. 8*D*, main effect of genotype: *F*_(1,32)_ = 20.50, *p* < 0.0001). A trend toward impaired plasticity was not statistically significant in this cohort (genotype × day interaction: *F*_(3,96)_ = 2.361, *p* = 0.0762). Similar to training with sequences, there was an increase in the magnitude of the positive-going component of the A_2_ VEP from Day 1 to Day 4 in *Ube3a^m−/p+^* mice, but no change in WT mice (Fig. 8*E*, two-way RM ANOVA: interaction: *F*_(1,33)_ = 11.60, *p* = 0.0017, post hoc WT Day 1 vs Day 4: *p* = 0.9025, *Ube3a^m−/p+^* Day 1 vs Day 4: *p* < 0.0001). In V1, plasticity occurred in both WT and *Ube3a^m−/p+^* mice (Fig. 8*F*,*G*, left, main effect of session: *F*_(1.383,20.75)_ = 34.67, *p* < 0.0001, right, main effect of session: *F*_(1.231,23.39)_ = 66.45, *p* < 0.0001) and there was no genotype difference (Fig. 8*H*, two-way RM ANOVA: main effect of genotype: *F*_(1,34)_ = 0.9498, *p* = 0.3366, interaction: *F*_(3,102)_ = 1.241, *p* = 0.2988).

Similarly, WT mice trained with individual familiar stimuli (Fig. 9*A*,*B*) showed stimulus-specific plasticity in ACC A_2_ latency (Fig. 9*C*, left, one-way RM ANOVA: main effect of session: *F*_(1.705,23.87)_ = 6.365, *p* = 0.0082), but there was no change in ACC A_2_ latency following training in *Ube3a^m−/p+^* mice (Fig. 9*C*, right, main effect of session: *F*_(1.858,22.30)_ = 2.425, *p* = 0.1145). A direct comparison between genotypes revealed an increase in A_2_ VEP latency across days in *Ube3a^m−/p+^* mice (Fig. 9*D*, main effect of genotype: *F*_(1,26)_ = 19.22, *p* = 0.0002). In this cohort, plasticity in the A_2_ VEP latency was not different by group (genotype × day interaction: *F*_(3,78)_ = 0.2868, *p* = 0.8348). In this group, there was an increase in the magnitude of the positive-going component of the A_2_ VEP from Day 1 to Day 4 in both WT and *Ube3a^m−/p+^* mice (Fig. 9*E*, two-way RM ANOVA: interaction: *F*_(1,26)_ = 2.075, *p* = 0.1617, main effect of session: *F*_(1,26)_ = 23.72, *p* < 0.0001, post hoc WT Day 1 vs Day 4: *p* = 0.0364, *Ube3a^m−/p+^* Day 1 vs Day 4: *p* = 0.0004). In V1, plasticity occurred in both WT and *Ube3a^m−/p+^* mice (Fig. 9*F*,*G*, left, main effect of session: *F*_(1.299,18.99)_ = 136.4, *p* < 0.0001, right, main effect of session: *F*_(1.968,23.62)_ = 102.7, *p* < 0.0001) and there was no genotype difference (Fig. 9*H*, main effect of genotype: *F*_(1,26)_ = 0.2909, *p* = 0.5942, interaction: *F*_(3,78)_ = 3.263, *p* = 0.0258).

### Familiar stimuli increase 1–30 Hz power in V1 but not in ACC

Transient changes in oscillatory power have been shown to encode visual stimulus familiarity in visual areas (Kissinger et al., 2018; Gao et al., 2021; Hayden et al., 2021; Tang et al., 2023). We hypothesized that oscillatory changes in ACC across training might contribute to the plasticity observed in the A_2_ VEP component. To address this question, we trained WT mice for 4 d using long-duration (1.5 s) single-orientation stimuli (Fig. 10*A*,*B*). As expected, training drove stimulus-specific potentiation of V1 VEP magnitude (Fig. 10*C*, one-way RM ANOVA: main effect of session: *F*_(1.844,27.67)_ = 53.06, *p* < 0.0001, post hoc A1 vs A4: *p* < 0.0001, A1 vs N4: *p* = 0.9864, A4 vs N4: *p* < 0.0001), coincident with a stimulus-specific increase in 1–30 Hz power in the stationary period following the VEP (Fig. 10*D*,*E*, main effect of session: *F*_(1.0696,15.34)_ = 44.42, *p* < 0.0001, post hoc A1 vs A4: *p* < 0.0001, A1 vs N4: *p* = 0.1312, A4 vs N4: *p* < 0.0001). In the same mice, training drove stimulus-specific plasticity in the latency of A_2_ VEP in ACC (Fig. 10*F*,*G*, main effect of session: *F*_(1.745,24.42)_ = 16.65, *p* < 0.0001, post hoc A1 vs A4: *p* = 0.0309, A1 vs N4: *p* = 0.0330, A4 vs N4: *p* < 0.0001). Unlike V1, there was no difference in 1–30 Hz power in ACC as a result of training (Fig. 10*H*,*I*, main effect of session: *F*_(1.225,17.16)_ = 2.845, *p* = 0.1040). These results suggest that experience-dependent plasticity in the A_2_ ACC VEP was not associated with detectable changes in oscillatory power.

## Discussion

In this work, we asked whether experience-dependent plasticity to visual sequences in mouse ACC reflects familiarity to the first stimulus or expectation of the second stimulus in a sequence. First, using long-duration stimuli, we demonstrated that ACC VEPs are biphasic (Fig. 2) and the A_2_ VEP component is the locus of plasticity (Fig. 3). With visual experience across days, A_2_ VEP latency becomes shorter, and critically, this plasticity reflects familiarity to stimulus A and not expectation of stimulus B (Fig. 6). In addition, we found that A_2_ VEP plasticity can be induced by training mice using a single orientation (Fig. 7). Overall, our results demonstrate that ACC “sequence plasticity” reflects familiarity rather than expectation and generalizes beyond sequences to other types of familiar stimuli. Finally, plasticity is impaired in the ACC of *Ube3a^m−/p+^* mice, and the locus of this impaired plasticity is the A_2_ VEP component (Figs. 5, 8, 9).

A central finding of this study is that ordered sequences, pseudorandom sequences starting with “A”, and exposure to “A” alone can drive plasticity in ACC VEP timing. The common feature of these protocols is repeated exposure to familiar stimulus “A” over days. Thus, we conclude that plasticity driven by each of these protocols reflects familiarity to stimulus “A”—and not, in the case of sequences, expectation of “B” following “A”. These protocols likely represent alternative approaches to drive a single, generalizable type of plasticity in ACC. This forces a revision of our understanding of “sequence plasticity,” a term that is insufficient to describe the broader phenomenon of experience-dependent plasticity in ACC VEP timing that is not specific to sequences. We propose to label this plasticity as “stimulus-specific response plasticity in timing” (SRPT). SRPT reflects familiarity to a single stimulus, but differs from V1 SRP because it is expressed by a change in VEP timing rather than VEP magnitude. Another subtle difference between ACC SRPT and V1 SRP is the induction protocol: while V1 SRP is typically induced using blocks of phase-reversing gratings, ACC SRPT requires that stimuli be separated by gray screens. A classical SRP protocol using phase reversals evoked low-magnitude ACC VEPs with an often undetectable A_2_ component (Fig. S7). We hypothesize that interleaved gray screen may enhance the stimulus salience, resulting in larger and more reliable ACC VEPs. This hypothesis aligns with prior studies demonstrating that ACC neurons are sensitive to visual salience (Hamm et al., 2021; Bastos et al., 2023).

For future work, we propose that a modified SRP protocol (as in Fig. 7) is best suited to evoke and study SRPT in ACC. While ordered and pseudorandom sequences were also effective at driving plasticity, additional stimuli beyond stimulus “A” are not necessary for SRPT induction. When induced by single-orientation stimuli, SRPT was specific to the trained stimulus: VEP latency on test day 4 was shorter to the trained stimulus than the novel stimulus (Figs. 7*D*, 9*C*) and similar to the latency on Day 1. We found a similar specificity when training with pseudorandom sequences (AXXX vs NXXX; Figs. 6*D*, 8*C*). These findings confirm that Day 1 responses reflect novelty of the visual stimulus, rather than aversiveness of seeing any visual stimulus for the first time. However, in experiments involving ordered sequences, VEP latencies were not statistically different on test day to “A” in familiar ABCD and “D” in novel DCBA (Figs. 1*D*, 3*F*, 5*D*). We hypothesize that these differences reflect familiarity: mice have had prior exposure to stimulus “D,” though in a different sequence position. However, when a test day stimulus was truly novel to the mouse, plasticity in all cases was specific to the familiar stimulus and did not generalize to the novel stimulus (Figs. 6*D*, 7*D*). Thus, we interpret Day 1 responses as reflecting novelty rather than a response to an aversive stimulus.

Prior studies of experience-dependent plasticity in mouse V1 provide a framework for hypothesizing about mechanisms and behavioral relevance of SRPT in ACC. Functionally, V1 SRP is associated with behavioral habituation to a familiar visual stimulus (Cooke et al., 2015), and recent work has described its synaptic and circuit-level mechanisms (summarized in Montgomery et al., 2021). In brief, V1 SRP requires activation of NMDA receptors (Cooke et al., 2015), requires sleep for consolidation (Aton et al., 2014), and is associated with oscillatory changes in the alpha/beta and gamma bands (Kissinger et al., 2018; Gao et al., 2021) likely driven by opposing changes in the activity of somatostatin + and parvalbumin + interneuron populations (Kaplan et al., 2016; Hayden et al., 2021). V1 sequence plasticity also requires sleep (Hosamane et al., 2024), though it differs mechanistically from SRP in other ways: for example, V1 sequence plasticity requires activation of M2 muscarinic acetylcholine receptors but not NMDA receptors (Gavornik and Bear, 2014; Sarkar et al., 2024), and V1 sequence plasticity but not V1 SRP is blocked by hippocampal lesion (Finnie et al., 2021). Our findings indicate that ACC SRPT occurs across days and not within a single session (Fig. 3*E*), suggesting a potential role for sleep similar to SRP and sequence plasticity in V1. However, unlike V1 SRP, ACC SRPT is not associated with oscillatory changes (Fig. 10). Future work will be needed to understand synaptic requirements for ACC SRPT induction, how ACC neuronal subpopulations adapt to visual experience, and specifically, to better understand the nature of the long-latency N2 VEP component in mouse ACC (“A_2_”) at a cellular level. We hypothesize that the plasticity expressed in ACC to sequence, pseudosequence, and nonsequence stimuli reflect the same underlying phenomenon and share underlying mechanisms.

Future work will be needed to understand whether and how latency changes in the VEP relate to behavior. V1 SRP is associated with behavioral habituation to familiar stimuli (Cooke et al., 2015), and we hypothesize that ACC SRPT may also reflect habituation. Because of behavioral states such as locomotion modulate visual processing and plasticity in V1 (Niell and Stryker, 2010; Fu et al., 2014; Vinck et al., 2015; Dadarlat and Stryker, 2017), it is possible that locomotion or attention may influence ACC VEPs. We did not address this question here, but prior work demonstrated that ACC VEP plasticity is expressed both when head-fixed mice are enclosed in a tube and moving on a running wheel (Sidorov et al., 2020). Future work on ACC SRPT should formally evaluate the potential role of attention and locomotion—a consideration that is especially important in *Ube3a^m−/p+^* mice, which display motor impairments on tests such as rotarod and open field (Sonzogni et al., 2018). More broadly, differences in how plasticity is expressed in ACC and V1 may relate to their respective roles in regulating behavior. ACC regulates visually guided behavior in multiple contexts (Kim et al., 2021; Norman et al., 2021; Broom et al., 2022; Peters et al., 2022; Cruz et al., 2023), and it will be important to understand if and how SRPT is linked to these broad roles in behavior.

Our prior work demonstrated that *Ube3a^m−/p+^* mice have impaired plasticity in ACC to familiar visual sequences (Sidorov et al., 2020). Here, we replicate and expand this finding using long-duration sequences, demonstrating that the latency of the A_2_ VEP component does not change with visual sequence training in *Ube3a^m−/p+^* mice (Fig. 5). We did not observe statistically significant genotype × day interactions using pseudorandom sequences or single orientations, but these studies did uncover broader disruptions in the A_2_ VEP in *Ube3a^m−/p+^* mice (Figs. 8, 9). For example, one feature of *Ube3a^m−/p+^* ACC responses that was conserved across all plasticity studies was a change in the magnitude of the A_2_ VEP following experience (Figs. 5*G*, 8*E*, 9*E*). These impairments appear specific to ACC: within mice, V1 plasticity driven by the same stimuli was normal. Together, these findings suggest that loss of *Ube3a* expression prevents ACC circuits from properly encoding familiarity. We hypothesize that impairments in *Ube3a^m−/p+^* mice could either be driven by changes in local ACC circuits or its inputs. ACC is agranular (Le Merre et al., 2021), differs in interneuron composition from V1 (Kim et al., 2017), and receives inputs from V1, V2M, and lateral posterior thalamus (Leow et al., 2022; Xue et al., 2022). Further work may evaluate whether these factors are involved in differences in SRPT in *Ube3a^m−/p+^* mice. Future work is also necessary to evaluate the behavioral consequences of impaired SRPT in *Ube3a^m−/p+^* ACC, though relevant behavioral impairments have been described in other contexts. First, *Ube3a^m−/p+^* mice have impaired performance in novel object recognition, suggesting a functional impairment in detecting familiarity (Godavarthi et al., 2012; Born et al., 2017). We hypothesize that this behavioral impairment could be linked to ACC activity, as ACC is known to contribute to encoding of this behavior (Weible et al., 2009). *Ube3a^m−/p+^* mice also have impairments in the visually guided five-choice serial reaction time task (Negron-Moreno et al., 2022), a known readout of ACC function (Koike et al., 2016).

We used VEPs to measure experience-dependent plasticity in ACC and V1—a technique with advantages and also several important limitations that limit interpretation of results. VEPs offer good temporal resolution, allow longitudinal assessment via chronic recordings, and have a long history in evaluating cortical function in awake mice (Gavornik and Bear, 2025). However, the VEP is a population-level signal rather than direct readout of spiking, lacks cellular resolution, and often has a low signal-to-noise ratio, requiring averaging across trials (Fig. S2). Our rationale was to enable a direct comparison with prior published work on “sequence plasticity” in ACC (Sidorov et al., 2020). VEPs were well suited to demonstrate that this plasticity reflects familiarity and generalizes beyond sequences. However, VEP limitations illustrate the need for alternative approaches to gain a better mechanistic understanding of SRPT in the ACC at the cellular and circuit levels. Our findings suggest that Ube3a loss disrupts typical experience-dependent changes in the A_2_ component of the VEP. Understanding the cellular processes driving this change will thus be critical to better understand how loss of Ube3a disrupts ACC circuits and how that might contribute to behavioral deficits.

Overall, this study demonstrated that sequence plasticity in mouse ACC reflects familiarity, and a similar form of plasticity (SRPT) can be evoked with single-orientation stimuli. Impaired ACC plasticity was observed in the *Ube3a^m−/p+^* mouse model of AS, and ACC SRPT could be a valuable approach for evaluating experience-dependent plasticity in related disorders.
